# Conditional CRISPR-Cas Genome Editing in *Drosophila* to Generate Intestinal Tumors

**DOI:** 10.3390/cells10113156

**Published:** 2021-11-13

**Authors:** Shivohum Bahuguna, Siamak Redhai, Jun Zhou, Tianyu Wang, Fillip Port, Michael Boutros

**Affiliations:** German Cancer Research Center (DKFZ), Division Signaling and Functional Genomics, BioQuant and Medical Faculty Mannheim, Heidelberg University, D-69120 Heidelberg, Germany; s.bahuguna@dkfz-heidelberg.de (S.B.); tianyu.wang@dkfz-heidelberg.de (T.W.); f.port@dkfz.de (F.P.)

**Keywords:** CRISPR, Cas9, tumors, BMP, Notch, JNK, intestinal stem cells, aging

## Abstract

CRISPR-Cas has revolutionized genetics and extensive efforts have been made to enhance its editing efficiency by developing increasingly more elaborate tools. Here, we evaluate the CRISPR-Cas9 system in *Drosophila melanogaster* to assess its ability to induce stem cell-derived tumors in the intestine. We generated conditional tissue-specific CRISPR knockouts using different Cas9 expression vectors with guide RNAs targeting the BMP, Notch, and JNK pathways in intestinal progenitors such as stem cells (ISCs) and enteroblasts (EBs). Perturbing Notch and BMP signaling increased the proliferation of ISCs/EBs and resulted in the formation of intestinal tumors, albeit with different efficiencies. By assessing both the anterior and posterior regions of the midgut, we observed regional differences in ISC/EB proliferation and tumor formation upon mutagenesis. Surprisingly, high continuous expression of Cas9 in ISCs/EBs blocked age-dependent increase in ISCs/EBs proliferation and when combined with gRNAs targeting tumor suppressors, it prevented tumorigenesis. However, no such effects were seen when temporal parameters of Cas9 were adjusted to regulate its expression levels or with a genetically modified version, which expresses Cas9 at lower levels, suggesting that fine-tuning Cas9 expression is essential to avoid deleterious effects. Our findings suggest that modifications to Cas9 expression results in differences in editing efficiency and careful considerations are required when choosing reagents for CRISPR-Cas9 mutagenesis studies. In summary, *Drosophila* can serve as a powerful model for context-dependent CRISPR-Cas based perturbations and to test genome-editing systems in vivo.

## 1. Introduction

The application of Clustered Regularly Interspersed Short Palindromic Repeats (CRISPR)—CRISPR-associated (Cas) systems for genomic studies has recently emerged as a powerful tool [1]. Initially discovered as a defense mechanism against invading viruses in bacteria and archaea, CRISPR-Cas has been adapted for genome engineering applications in many organisms [1,2]. In the most widely used CRISPR system, the RNA-guided endonuclease Cas9 is targeted to specific DNA sequences to create double-strand breaks (DSBs) by a short guide-RNA (gRNA) sequence containing several nucleotides that are complementary to the target gene of interest [3,4]. The only limitation in choosing a target site for the prototypical Cas9 from *Streptococcus pyogenes* is that it must be adjacent to a protospacer adjacent motif (PAM) site of the sequence NGG. Cleavage of the target site and subsequent DSBs are repaired by the cellular DNA repair pathways, which includes the error prone non-homologous end joining pathway (NHEJ pathway) and the more precise homology-directed repair (HDR). However, cellular repair pathways frequently lead to the insertion or deletion of nucleotides at the target site, often causing loss of function (LOF), or sometimes, gain of function mutations [5,6]. Indeed, in recent times, CRISPR-Cas9 has been used in various fields including cancer genetics [6].

Over the past century, *Drosophila melanogaster* has emerged as an important model organism for biological studies due in large part to the vast number of genetic tools developed in this organism [7,8]. Noticeably, the GAL4/UAS system has emerged at the forefront of these efforts [9]. GAL4 can bind to upstream activating sequence (UAS) and activate expression of downstream genes. By coupling GAL4 expression with defined tissue-specific promoters, UAS-driven genes can be restricted to a desired location in the fly. Additionally, temporal control of this system can be achieved by introducing the GAL80^ts^ element, which binds the GAL4 transcriptional activation domain and prevents its activity at a permissive temperature of 18 °C [10]. However, at the restrictive temperature of 29 °C or above, GAL80^ts^ no longer binds and represses GAL4, therefore allowing UAS-driven gene expression. Interestingly, CRISPR-Cas technology has been used in *Drosophila melanogaster* for heritable germline mutagenesis. By driving Cas9 and gRNA with the binary GAL4/UAS system, biallelic mutations can be achieved in a tissue-specific manner [11,12,13]. 

Modifications to the CRISPR-Cas system in *Drosophila* have been made in the past to improve its editing efficiency [14]. Initially, both microinjection of Cas9 and single-guide RNAs (gRNA) in the form of either plasmids, in vitro transcribed RNA or ribonucleoprotein particles, and stable integration of DNA constructs into the genome were used for mutagenesis, albeit with different outcomes [15,16]. Moreover, editing rates were also observed to change when different integration sites of the CRISPR-Cas elements were used or by manipulating cis-regulatory elements [17,18]. Interestingly, it has been observed that very high expression levels of Cas9 resulting from some of the original expression constructs such as *UAS-Cas9.P1* can be toxic to cells [13,17]. Attempts at reducing toxicity by expressing Cas9 from plasmids tuned to lower expression such as *UAS-Cas9.P2* resulted in some success, but some toxicity remained [11]. More recently, by using upstream open reading frames (uORF) of different lengths, it has been possible to titrate Cas9 to more optimal levels, and constructs such as the *UAS-u^M^Cas9* have been shown to be less toxic [11]. 

The intestinal tract of *Drosophila melanogaster* has been extensively used for studies into metabolism, regeneration, and cancer biology, and parallels can be drawn with mammalian systems due to its functional and anatomical similarities [19]. The *Drosophila* midgut is maintained by proliferating intestinal stem cells (ISCs) that give rise to differentiating enteroblast progenitors (EBs) that can further mature into absorptive enterocytes (ECs) or secretory enteroendocrine cells (EEs) [19]. Previous studies have highlighted numerous signaling pathways that are involved in aspects of ISC division, differentiation, and tissue damage. Of note, conserved pathways such as Notch and BMP/Dpp signaling have been reported to control various aspects of differentiation and proliferation [20]. To date, CRISPR-Cas9 studies in the *Drosophila* intestine have been limited [21,22]. Here, we performed an analysis of two popular Cas9 expression constructs (*Cas9.P2 and u^M^Cas9*) for their ability to specifically alter ISCs/EBs biology. We targeted the BMP, Notch, and JNK pathways with two gRNAs against *Mothers against dpp* (*Mad*), *neuralized* (*neur*), *Notch*, and *hemipterous* (*hep*) and assessed parameters such as ISC/EB number, mitotic index, tumor incidence, and morphological changes to the intestine. We found that targeting these pathways induces regional proliferation of stem cells and tumor formation, albeit to different extents. Importantly, continuous expression of *Cas9.P2* in this tissue was deleterious to stem cells, but regulating *Cas9.P2* expression with temporal control or using *u^M^Cas9* allowed efficient mutagenesis to take place.

## 2. Material and Methods

### 2.1. Fly Stocks

The following lines were used: esg-Gal4; tub-Gal80^ts^, UAS-GFP (esg^ts^) [23], esg-Gal4, tub-Gal80^ts^, UAS-GFP; UAS-Cas9.P2 (esg^ts^ > Cas9.P2) [11], esg-Gal4, tub-Gal80^ts^, UAS-GFP; UAS-u^M^Cas9 (esg^ts^ > u^M^Cas9) [11], P{hsFLP}1, y^1^ w^1118^; P{HD_CFD01184}attP40-Notch gRNA^2X^ (VDRC 341922), P{hsFLP}1, y^1^ w^1118^; P{HD_CFD00651}attP40-Mad gRNA^2X^ (VDRC 341570), P{hsFLP}1, y^1^ w^1118^; P{HD_CFD01179}attP40-neur gRNA^2X^ (VDRC 341917), P{hsFLP}1, y^1^ w^1118^; P{HD_CFD01377}attP40-hep gRNA^2X^ (VDRC 342022). All gRNA lines were previously made [11].

### 2.2. CRISPR/Cas9 Mutagenesis 

Flies were kept on a standard cornmeal/agar diet with a 12 h:12 h light:dark cycle. One L of standard diet contained 44 g sugar syrup, 80 g malt, 80 g corn flour premium G750, 10 g soy flour, 18 g yeast, 2.4 g methly-4-hyroxybenzoate, 6.6 mL propionic acid, 0.66 mL phosphoric acid, and 8 g agar [22]. Flies requiring adult expression of transgenes were initially raised at 18 °C (permissive temperature) and switched to 29 °C (restrictive temperature) post eclosion for specific durations. For experiments involving *UAS-Cas9.P2*, newly eclosed flies were shifted to 29 °C for 10 days and 18 °C for 30 days before being switched back to 29 °C for one day. For experiments involving *UAS-u^M^Cas9*, newly eclosed flies were shifted to 29 °C for 20 days. Flies were transferred into fresh food once every two days to avoid fungal infection. Mated females were used for all experiments. 

### 2.3. Immunostaining and Image Acquisition

Adult flies were anesthetized on a CO_2_ pad and female flies were dissected in 1 × PBS. Whole guts were transferred onto poly-lysine slides and fixed with 4% formaldehylde (28908, VWR diluted in PBS) for 30 min. The intestines were then washed with 1 × PBST (1 × PBS + 0.1% Triton X-100 (T8787-250 mL, Sigma, Darmstadt, Germany)) and blocked with 1× PBST + 1% BSA (1062, Gerbu, Heidelberg, Germany). The tissues were incubated overnight with the primary antibody at 4 °C and washed with 1 × PBST. Fluorescently labelled secondary antibodies were diluted in 1 × PBST (final concentration 1:3000) and incubated at room temperature for 1.5 h. The midguts were then mounted in Vectashield containing DAPI (H-1200, Linaris, Burlingame, CA, USA). Staining of experimental and control samples was carried out on the same slide for direct comparisons. The same confocal settings (e.g., laser power, gain, and pinhole) were applied to both experimental and control groups. Primary antibodies were rabbit anti-pH3 (1:500, cell signaling 9701L), mouse anti-Cas9 (1:500 Cell signaling 14,697 s), and secondary antibodies were chicken anti rabbit AF594 (1:3000, A21442, Invitrogen, Waltham, MA, USA) and donkey anti mouse AF594 (1:3000, A21203, Invitrogen, Waltham, MA, USA).

### 2.4. Quantification of Midgut Mitosis 

Dividing cells were marked with Phospho-Histone H3 staining (pH3) antibody and mitotic cells were counted in the anterior and posterior intestine using a Leica SP8 confocal microscope with a 40× objective. Representative images were taken using a Leica SP8 confocal microscope. Images were analyzed, processed, and compiled using Prism 8, Fiji [24], and Inkscape.

### 2.5. Quantification of GFP Positive Cells

Whole z-stacked images of the entire anterior (R2) and posterior (R4 and R5) midgut regions were taken using either a Leica SP5, SP8, or a Nikon A1 confocal microscope. The progenitor cells labelled with GFP (*esg-GAL4, tub-Gal80^ts^, UAS-GFP*) and nuclei of all intestinal cells labelled with DAPI were counted using an ImageJ macro (developed by Dr Damir Krunic from the DKFZ imaging facility). This function segments the intestines and counts the total number of DAPI^+^ nuclei and GFP^+^ cells. We used the following formula: (GFP^+^/DAPI^+^) × 100, to generate the percentage of GFP^+^ cells in the region of interest. Images were analyzed, processed, and compiled using Prism 8, Fiji [24], and Inkscape.

### 2.6. Measurement of Midgut Length and Diameter

Guts were dissected, placed on a polylysine-coated slide and fixed with 4% formaldehyde (28908, VWR diluted in PBS) for 30 min. Images of the entire midgut were taken using a Zeiss stereo microscope. Lines were drawn from the base of the proventriculus until the midgut–hindgut boundary using the free hand line tool in Fiji. The length of lines was measured and plotted in Prism 8. Full length representative images of different genotypes were taken using a Leica SP8 microscope on a 20× objective lens and stitched together using the pairwise stitching tool from Fiji. For gut diameter measurements, a z-projected view of the intestine was used, and a line was drawn perpendicular to the gut and measured with Fiji. Images were compiled in Inkscape.

### 2.7. Tumor Incidence 

The tumor incidence was determined by counting the number of midguts with tumors in the mutant and control group. Tumors were defined as clusters of GFP positive cells with small DAPI nuclei. Representative images are shown for what we considered a tumor in Figure 4A. The percentage of tumor bearing flies were obtained from 2–3 independent experiments and the results were pooled to generate the related bar figure.

### 2.8. Statistical Analysis

All data were analyzed using Prism 8 software (GraphPad Software, San Diego, CA, USA). For comparisons between two groups, a student *t* test was performed and for more than two groups, an ordinary one-way ANOVA test was used with multiple comparisons between groups. An outlier test was performed on datasets to remove any outliers. Significance values are represented as follows: ns: not significant, * *p* < 0.05, ** *p* < 0.01, *** *p* < 0.001, **** *p* < 0.0001. All data points represent one animal and are marked with individual dots, data are shown using either a heatmap, stacked bar charts, or boxplots.

## 3. Results 

### 3.1. Cas9 Expression in the Drosophila Midgut

**Figure 1:**
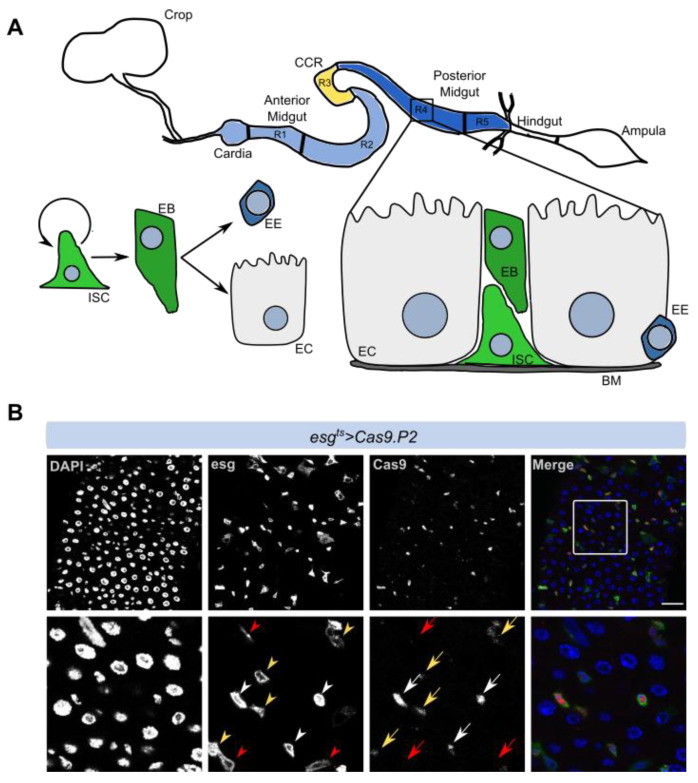
Digestive tract and intestinal cell types in *Drosophila melanogaster.* (**A**) Schematic representation of the intestine, which contains different regions (R1–R5) that house progenitor cells such as intestinal stem cells (ISCs) and enteroblasts (EBs), which can differentiate into enterocytes (ECs) or enteroendocrine cells (EEs). (**B**) ISCs/EBs are dispersed throughout the midgut and can be marked with *escargot (esg > GFP)* (arrowheads). Notice that *esg > GFP* expression is variable (compare white and yellow arrowheads). Expressing *Cas9.P2* using the *esg^ts^* system and staining against Cas9 (Red) reveals that this construct is translated into protein in ISCs/EBs (arrows). Notice that Cas9 expression is variable (compare white and yellow arrows) and some *esg > GFP* cells lack detectable Cas9 expression (red arrowheads and red arrows). Scale bar: 30 µm.

The *Drosophila* midgut is compartmentalized and comprises several cell types including ISCs that can divide and give rise to EBs, which further differentiate into secretory enteroendocrine cells or absorptive enterocytes (Figure 1A). We used the *esgGal4, tub-Gal80^ts^, UAS-GF*P (*esg^t^*^s^) system to target progenitor cells including intestinal stem cells and enteroblasts (ISCs/EBs) in adult flies, circumventing defects that might arise due to the expression of genetic constructs during development (see Materials and Methods). We first crossed two Cas9 constructs (*Cas9.P2* and *u^M^Cas9*) into the *esg^t^*^s^ fly in order to stably express *Cas9* during adulthood using temporal control (*esg^ts^ > Cas9.P2* or *esg^ts^ > u^M^Cas9*). While *Cas9.P2* has previously been shown to have strong expression, *u^M^Cas9* harbors an upstream opening reading frame, which dampens its expression, thus allowing us to perform mutagenesis in different conditions. To assess whether our *esg^ts^* system expresses Cas9 specifically in progenitor cells, we induced its expression and performed antibody staining against Cas9. We found that in flies expressing *esg^ts^ > Cas9.P2,* Cas9 staining was detectable in ISCs/EBs, albeit with different degrees, which likely arises as a result of variable Gal4 expression (Figure 1B). In flies expressing *esg^ts^ > u^M^Cas9,* Cas9 was not detectable by immunostaining in ISCs/EBs, supporting the model that *u^M^Cas9* is expressed at a lower level compared to *Cas9.P2* (data not shown). In our previous study, we showed that Cas9 immunostaining is detectable with *u^M^Cas9*, but at a lower level when compared to *Cas9.P2* in the wing disc [11]. In summary, high expression of *Cas9* in ISCs/EBs resulted in detectable Cas9 protein in these cell types. 

### 3.2. Defining Temporal Parameters for Cas9 Editing 

**Figure 2:**
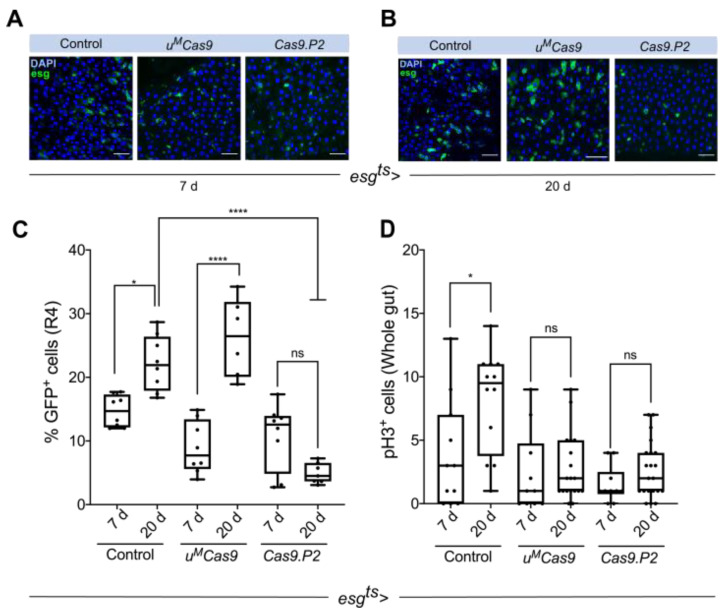
Continuous expression of *Cas9.P2* is deleterious to ISCs/EBs. (**A**) ISCs/EBs in control animals displayed an age-dependent increase in their numbers (A; 7 days, B; 20 days, see quantifications in panel C). (**B**) Continuous expression of *Cas9.P2*, but not *u^M^Cas9*, for 20 days noticeably reduced ISCs/EBs. (**C**) Quantifications of ISCs/EBs numbers at seven days and 20 days in animals either expressing no *Cas9*, *u^M^Cas9*, or *Cas9.P2*. Notice that in both the control animals and those expressing *u^M^Cas9*, ISCs/EBs significantly increased from seven days to 20 days. (**D**) The number of mitotic cells increased in the whole gut of the control animals from seven days to 20 days, but this could not be seen in animals continuously expressing *Cas9.P2* at these timepoints. Scale bar: 30 µm. * *p* < 0.05, **** *p* < 0.0001.

To test if continuous *Cas9* expression is deleterious to ISCs/EBs, we expressed both *Cas9* constructs for different durations and quantified ISCs/EBs numbers. Previous reports suggest that the number of ISCs/EBs increases with age [25]. We used two different timepoints (seven days and 20 days) to assess whether ISCs/EBs numbers were altered in the R4 region of the intestine when different *Cas9* transgenes were expressed. In the control animals, we observed a significant increase in ISCs/EBs from seven days to 20 days (Figure 2A–C). Although continuous expression of *Cas9.P2* did not alter the number of ISC/EBs at seven days (Figure 2A,C), it did significantly reduce their numbers at 20 days, suggesting that prolonged expression of *Cas9.P2* may be deleterious to these cells (Figure 2A–C). We also observed that continuous expression of *Cas9.P2* caused ISCs/EBs to appear more rounded (Figure 2B). Interestingly, no such effects were observed for *u^M^Cas9*, neither at seven days nor 20 days, and ISCs/EBs increased in an age-dependent manner similar to the control animals, suggesting that this construct is well tolerated in the intestine (Figure 2A–C). 

To complement these results, we performed the same time-course experiments in the control and *esg^ts^ > Cas9.P2* flies and stained the intestines with phospho-histone 3 (pH3), which marks mitotically active cells. Whilst mitotic cells in control flies increased from seven days to 20 days, this effect was abrogated in flies continuously expressing *Cas9.P2* for 20 days (Figure 2D). Surprisingly, we did not observe an increase in the number of mitotic cells in flies expressing *esg^ts^ > u^M^Cas9* during these timepoints, suggesting that *u^M^Cas9* also has some deleterious effects on actively dividing cells. In conclusion, continuous expression of *Cas9.P2* prevents an increase in ISC/EB and pH3^+^ cells, while expressing *u^M^Cas9* led to an age-dependent increase in ISCs/EBs but not pH3^+^ cells.

### 3.3. Cas9.P2^on/off^ and u^M^Cas9^on^ Are Effective at Inducing Intestinal Tumors 

**Figure 3:**
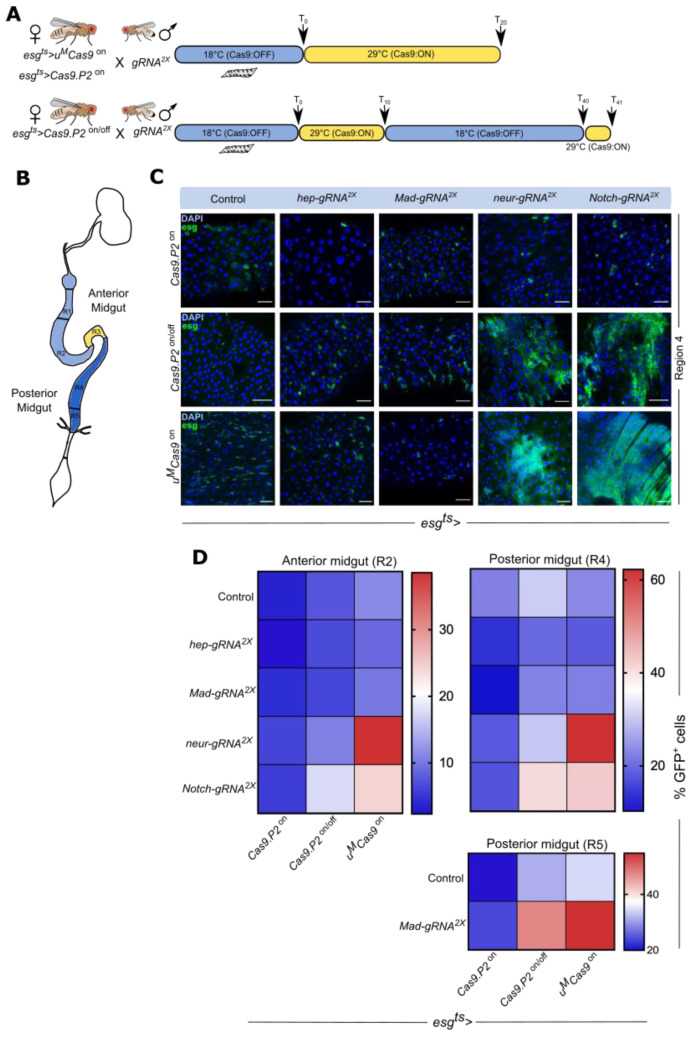
Both Cas9.P2^on/off^ and u^M^Cas9^on^ are effective at inducing intestinal stem cell derived tumors. (**A**) Schematic representation of the timepoints used for experiments. Female flies expressing either *Cas9.P2^on^* or *u^M^Cas9^on^* were crossed to male flies expressing different *gRNAs*. The cross was maintained at 18 °C and the progeny were raised under these conditions during their development. After adult progeny emerged (T_0_), they were shifted to 29 °C continuously for 20 days to induce the *esg^ts^* expression system, which allows for both *Cas9* and *gRNAs* to be expressed in ISCs/EBs. Since high expression of *Cas9.P2* is toxic, we also devised an on/off strategy (*Cas9.P2 ^on/off^*), whereby adult progeny were initially shifted to 29 °C for 10 days (T_10_), then to 18 °C for 30 days (T_40_) and finally at 29 °C for one day (T_41_). (**B**) Schematic representation of the intestine with different regions highlighted. (**C**) Confocal images of the R4 regions of the intestine in flies expressing *Cas9* and *gRNAs* against *hep*, *Mad*, *neur*, and *Notch*. Notice that for *Cas9.P2^on/off^* and *u^M^Cas9^on^* with *gRNA* against *neur* and *Notch* resulted in massive proliferation of ISCs/EBs. (**D**) Heatmap showing the average percentage of GFP^+^ ISCs/EBs after using different Cas9 enzymes for perturbations. Scale bar: 30 μm.

We set out to test the editing efficiencies of *Cas9.P2* and *u^M^Cas9* by targeting members of several conserved signaling pathways known for their role in tumor development. These include the BMP, Notch, and JNK cascades [21,26,27]. We assessed both the anterior and posterior portions of the intestine to test for regional differences in the number of ISC/EB derived tumors. Since continuous expression of *Cas9.P2* is toxic to ISCs/EBs, we designed a temperature shift regiment to tune its expression and reduce toxicity. We found that switching *Cas9.P2* expression on for 10 days and then off for 30 days was sufficient, allowing for efficient editing to take place (Figure 3A,B). At the end of this timepoint, we switched the system on for one day in order to express GFP and visualize ISCs/EBs (Figure 3A, see Materials and Methods). We designated this as *Cas9.P2^on/off^*. For *u^M^Cas9^on^*, we found 20 days of expression to be sufficient for mutagenesis and also included *Cas9.P2 ^on^* at this timepoint for direct comparison (Figure 3A,B). 

We next used two transgenic *gRNAs* (*gRNA^2x^*) from the Heidelberg CRISPR Fly Design Library [11] to target the tumor suppressors *Mad*, *Notch*, and *neur* with either *Cas9.P2* (*esg^ts^ > Cas9.P2*) or *u^M^Cas9* (*esg^ts^ > u^M^Cas9*) and dissected the intestine of the progeny at the timepoints above-mentioned (Figure 3A). We also used *gRNA^2x^* against *hep* as a control since mutating this gene has previously been shown to inhibit age-dependent increase of ISCs/EBs [25]. Continuous expression of *Cas9.P2* (*esg^ts^ > Cas9.P2^on^*) failed to show observable changes in ISC/EB numbers when perturbing either *Mad*, *Notch*, or *neur*, suggesting either low-efficiency mutagenesis or suppression of proliferation normally associated with such mutations in these genes by Cas9 mediated toxicity (Figure 3C,D and Figure S1A). However, adopting an on/off regiment for *Cas9.P2* (*esg^ts^ > Cas9.P2^on/off^*) increased ISC/EB numbers in the R4 region when either *Notch* or *neur* was perturbed and decreased ISCs/EBs numbers when using *gRNA^2x^* against *hep* (Figure 3C,D and Figure S1B). Moreover, perturbations of *Notch* with *Cas9.P2^on/off^* also increased ISC/EB numbers in the R2 region, highlighting regional differences when compared to *neur* (Figure 3D and Figure S1B). These changes have been previously observed in LOF studies of these genes, confirming the specificity of the gRNAs used [22,26,27]. Interestingly, *gRNA^2x^* against *Mad* failed to increase ISC/EB proliferation in the R2/R4 region, but did increase their numbers in the R5 region, highlighting regional specificity (Figure 3D and Figure S2A,B). In flies continuously expressing *u^M^Cas9* (*esg^ts^ > u^M^Cas9^on^*) with *gRNA^2x^* targeting *neur* and *Notch*, we observed a significant increase in ISC/EB proliferation in both R2/R4 regions, although the effect for *Notch* in the R2 region was more modest when compared to *neur* (Figure 3C,D and Figure S1C). Similar to *Cas9.P2^on/off^*, *u^M^Cas9^on^* showed regionalized proliferation of ISCs/EBs in the R5 region after mutating *Mad* (Figure 3D and Figure S2A,B), although this construct did not change ISC/EB numbers when *gRNA^2x^* against *hep* was used (Figure 3C,D). In summary, both *u^M^Cas9^on^* and *Cas9.P2^on/off^* are effective tools to induce mutations in the intestine and for studying tumorigenesis. 

### 3.4. Mutating Tumor Suppressors Increases Mitotic Cells and Tumor Incidence 

To complement the findings above, we assayed mitotic activity by staining the intestine with pH3, which marks mitotic cells. Both *Cas9.P2^on/off^* and *u^M^Cas9^on^,* but not *Cas9.P2^on^*, increased the number of mitotic cells after targeting either *neur*, *Notch*, or *Mad* (Figures S3C,D and S4A–C). Indeed, we also observed regional changes in pH3 numbers, with perturbations of Mad resulting in higher pH3^+^ cells in the posterior R5 portion of the intestine compared to the anterior (Figures S3B,C and S4B,C). When targeting *Notch* or *neur* with either *Cas9.P2^on/off^* and *u^M^Cas9^on^*, an increase in pH3^+^ cells was observed in both the anterior and posterior regions (Figure S3C,D), suggesting that more ISCs/EBs are mitotically active. 

**Figure 4:**
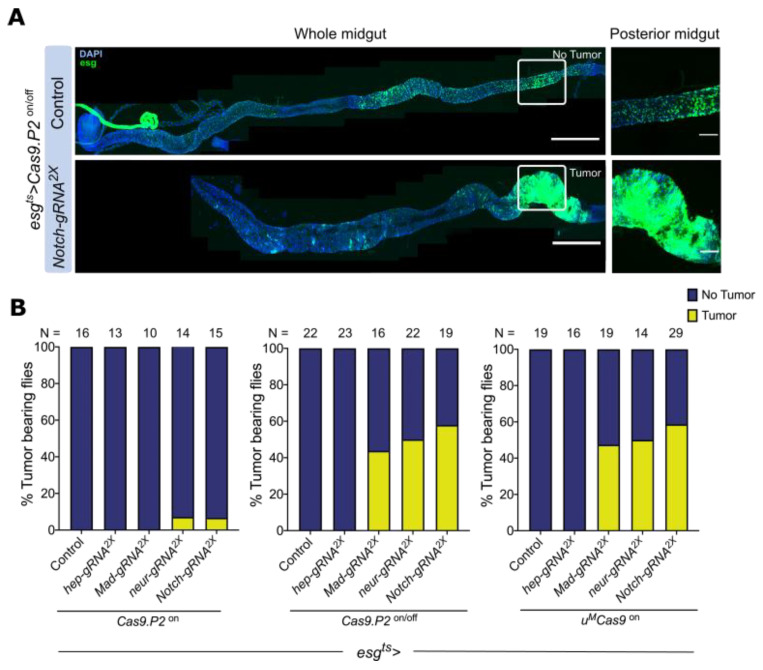
Tumor incidence rate increases after perturbing tumor suppressors. (**A**) Intestinal tumors can be observed after perturbing the Notch pathway; notice the cluster of GFP^+^ cells accumulated in the posterior midgut (insert). (**B**) Quantification of the number of tumor bearing flies after perturbing the BMP, Notch, and JNK pathways. Both *Cas9.P2^on/off^* and *u^M^Cas9^on^* increase the percentage of tumor bearing flies when *gRNAs* were used to target *Mad*, *neur*, or *Notch*, while no such effects were seen with *Cas9.P2^on^*. Scale bar: 350 µm, insert—60 µm.

Mutations in tumor suppressors such as *Notch*, *neur*, or *Mad* in progenitor cells are known to cause intestinal tumors [22,26]. To determine how effective *Cas9.P2* and *u^M^Cas9* are in inducing intestinal tumors, we quantified the tumor incidence rate in the population. We scored intestinal tumors as areas of dense GFP^+^ cells containing small DAPI^+^ nuclei (Figure 4A). Both *Cas9.P2^on/off^* and *u^M^Cas9^on^* significantly increased the percentage of tumor bearing flies when either *Mad*, *Notch*, or *neur* was mutated (Figure 4B), highlighting the effectiveness of these tools for tumorigenesis studies.

### 3.5. Mutating Tumor Suppressors Changes Intestinal Morphology 

**Figure 5:**
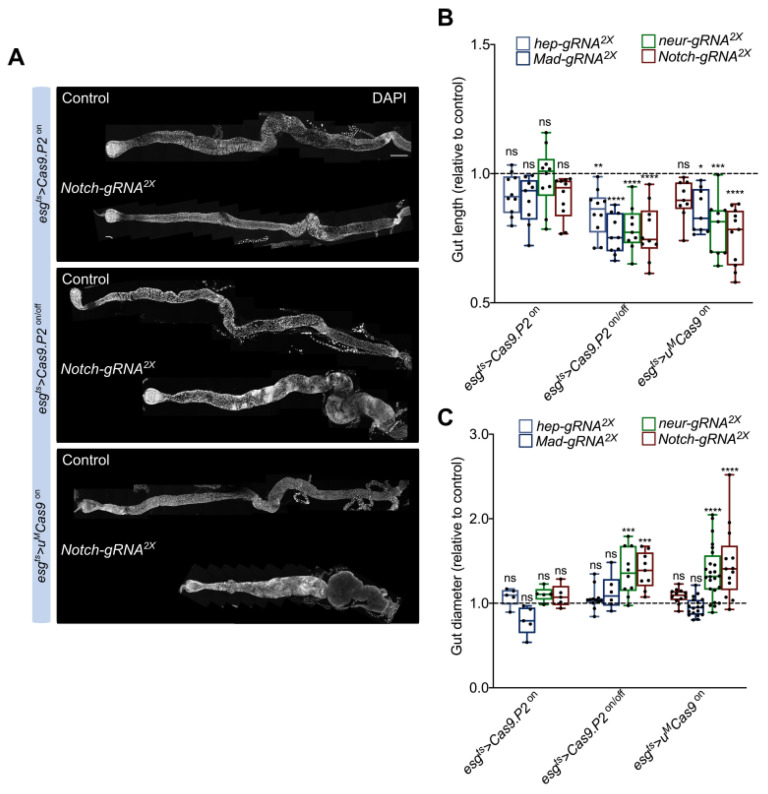
Intestinal morphology changes after perturbing BMP, Notch, and JNK signaling. (**A**) The intestine was imaged and stitched together to visualize morphological changes after perturbing the BMP, Notch, and JNK pathways. Notch perturbations are used as an example to depict intestinal length shortening. Notice clusters of DAPI^+^ nuclei when using *Cas9.P2^on/off^* or *u^M^Cas9^on^* with *Notch gRNA^2x^*. (**B**) Quantification of intestinal length relative to the control. Intestinal length was shorter when the BMP, Notch, and JNK pathways were perturbed using *Cas9.P2^on/off^* or *u^M^Cas9^on^*, but not with *Cas9.P2^on^*. (**C**) quantification of the diameter (R4 region) of the intestine relative to the control. Gut diameter increased when Notch signaling were perturbed using *Cas9.P2^on/off^* or *u^M^Cas9^on^*, but not with *Cas9.P2^on^*. Scale bar: 350 µm. * *p* < 0.05, ** *p* < 0.01, *** *p* < 0.001, **** *p* < 0.0001.

To gauge the physiological consequences of inducing intestinal tumors, we focused on morphological changes to the intestine. We quantified intestinal length after perturbing either BMP, Notch, or JNK signaling since previous studies reported that intestinal length is associated with diseases such as short bowel syndrome, which causes malnutrition, dehydration, and weight loss [28]. We observed a shortening of the intestine when either BMP (*Mad gRNA^2x^*) or Notch (*Notch gRNA^2x^, neur gRNA^2x^*) derived tumors were formed using either *Cas9.P2^on/off^* and *u^M^Cas9^on^* (Figure 5A,B). Moreover, Notch induced tumors also led to an accumulation of small nuclei in the epithelium (DAPI^+^ cells), which has previously been reported to be EEs and ISCs/EBs (Figure 5A) [26]. Indeed, changes in cell composition and cell death may contribute toward the observable changes in gut length. We also observed non-significant changes in intestinal length using *Cas9.P2^on^* with *gRNA^2x^* against *hep*, *Mad*, *neur*, or *Notch*, confirming that toxicity of *Cas9.P2^on^* outweighs its editing capabilities. We further quantified the diameter of the intestine in the R4 region and found that gut diameter increased when Notch signaling were perturbed using *Cas9.P2^on/off^* or *u^M^Cas9^on^*, but not with *Cas9.P2^on^*. In summary, we found that CRISPR induced tumorigenesis in the intestine shortens its overall length and increases the diameter of the gut, which likely impacts other physiological parameters. 

## 4. Discussion

Genome editing by CRISPR-Cas9 technology has greatly advanced our ability to generate mutations in vivo in a number of organisms. In the present study, we reported a simple and effective method for conditional tissue-specific CRISPR mutagenesis in the *Drosophila* intestine. As proof of principle, we used the Gal4/UAS system in combination with Gal80^ts^ to express *Cas9* in adult *esg+* intestinal progenitor cells such as ISCs and EBs, combined with two gRNAs targeting the tumor suppressors *Notch*, *neur*, and *Mad*. These perturbations resulted in regionalized proliferation of ISCs and tumor formation. Moreover, we showed that continuous high expression of Cas9 is deleterious to ISCs and that fine tuning its levels using different transgenes (*Cas9.P2* and *u^M^Cas9*) or applying various temporal strategies results in mutagenesis and tumorigenesis in the intestine. 

### 4.1. Comparison of Intestinal-Specific CRISPR-Cas9 Systems

RNA interference (RNAi) based LOF studies have been widely used to investigate tissue-specific functions for genes in various model organisms including *Drosophila*. Due to years of efforts, several in vivo RNAi transgenic libraries have been generated and used successfully in LOF studies [29,30,31,32,33,34]. These reagents have been made publicly accessible through various *Drosophila* stock centers (e.g., GD/KK libraries, TRiP library) [29,35,36]. However, the RNAi associated techniques have certain limitations such as off target effects and variable efficiencies. 

As an alternative, CRISPR-Cas9 approaches have been applied for in vivo LOF studies. Our recently developed large-scale transgenic CRISPR-Cas9 systems, which express multiple gRNAs or genetically modified Cas9 enzymes, has greatly improved gene editing [11,13,17]. We previously compared RNAi and gRNA targeting Notch directly in the intestine, and showed that while *Notch-RNAi* generated intestinal tumors in only females, *Notch-gRNA^x2^* resulted in tumor formation in both male and female flies, suggesting that using CRISPR leads to more robust mutagenesis and intestinal tumor phenotypes [11]. The efficiency of CRISPR-Cas relies not only on the design of the gRNA and the sgRNA expression vector, but also on the extent of Cas9 expression in different tissue or cell type of interest [11,13,17]. Previous studies have demonstrated that CRISPR induced mutagenesis typically has efficiencies in the range of 25–100% [11,17]. Indeed, estimating the number of mutated cells in vivo using CRISPR-Cas can be cumbersome if antibodies against the targeted gene do not exist or expression of the targeted gene is not stopped. Testing the editing efficiency of the gRNAs used in this manuscript at the single cell level will be an avenue for future research. 

Indeed, due to the vast array of genetic tools developed in *Drosophila* including different CRISPR tools, this model organism is unmatched in its ability to perform tissue-specific and cell-type specific mutagenesis. In this study, we compared the CRISPR targeting efficiency of different *Cas9* transgenes and found that *u^M^Cas9* yielded a stronger intestinal tumorigenic phenotype than *Cas9.P2^on^* when combined with *gRNAs* against tumor suppressors. We noticed that high expression of *Cas9* protein had a negative impact on stem cell activity in the intestine, which is consistent with previous reports in other tissues or organisms [11,37,38]. For this reason, we developed an on/off expression strategy for *Cas9.P2* by using a temperature sensitive system. As a result, *Cas9.P2^on^*^/off^ has significantly enhanced intestinal tumor phenotypes when combined with gRNAs targeting the BMP and Notch pathways. 

### 4.2. Effects of High Cas9 Expression and Outlook on Applications of CRISPR Fly Models

Our study demonstrates the importance of regulating Cas9 expression for effective mutagenesis. In agreement with previous studies, we showed that high expression of Cas9.P2 prevent age-associated increase in ISCs [13,17]. Indeed, continuous high expression of this construct also failed to recapitulate loss of function phenotypes seen when Notch and BMP signaling is perturbed in the intestine. Although we did not address how toxicity arises when Cas9.P2 is continuously expressed, we found that titrating its expression level by temporarily regulating its expression or using the genetically modified u^M^Cas9, results in robust tumor phenotypes developing after disrupting the Notch or BMP pathway. Future studies will be aimed at better understanding the causes of toxicity associated with high Cas9 expression. 

Conserved signaling pathways such as Notch, BMP, Wnt, Ras, and JAK/STAT are essential for intestinal homeostasis and their dysregulation can result in intestinal tumorigenesis [21,25,39,40,41,42,43,44,45,46]. Nearly 75% of the genes responsible for human disease have a *Drosophila* homolog [47,48]. Recent efforts at building fly models of human cancers have allowed drug screens to take place, subsequently leading to patient studies [46,47,49,50]. With the precision of CRISPR-Cas in editing the genome, attempts at making patient specific mutations in fly using this strategy combined with drug screening could facilitate a more personalized approach for treatment. Additionally, with an in vivo fly model, other factors such the tumor microenvironment, host–microbe interaction, and changes in organelle/cellular trafficking pathways could also be taken into consideration in order to gain a better mechanistic understanding of tumorigenesis [21,22,51]. In summary, our study lends future support to using CRISPR-Cas editing to study tumor biology in *Drosophila melanogaster,* which serves as an important model organism. 

## Data Availability

Not applicable.

## Supplementary Materials

The following are available online at https://www.mdpi.com/article/10.3390/cells10113156/s1, Figure S1. Boxplot representation of GFP^+^ cells after perturbation of the BMP, Notch and JNK pathways. Figure S2. Quantification of GFP+ cells in the R5 region when Mad is perturbed with different Cas9 enzymes. Figure S3. Cas9.P2^on/off^ and u^M^Cas9^on^ mediated perturbations of tumor suppressors increase the number of mitotic cells in the intestine. Figure S4. Boxplot representation of pH3^+^ cells after perturbation of the BMP, Notch and JNK pathways.
