# Supplementary material for: Conditional CRISPR-Cas Genome Editing in Drosophila to Generate Intestinal Tumors
Source: Cells. 2021 Nov 13;10(11):3156. doi: 10.3390/cells10113156 (PMC8620722; doi:10.3390/cells10113156)
Supplement: Supplementary file 1 [file cells-10-03156-s001.zip › cells-1418621-supplementary.pdf]

Supplementary Materials: The following are available online at www.mdpi.com/xxx/s1, Figure S1: title, Table S1: title, Video S1: title.

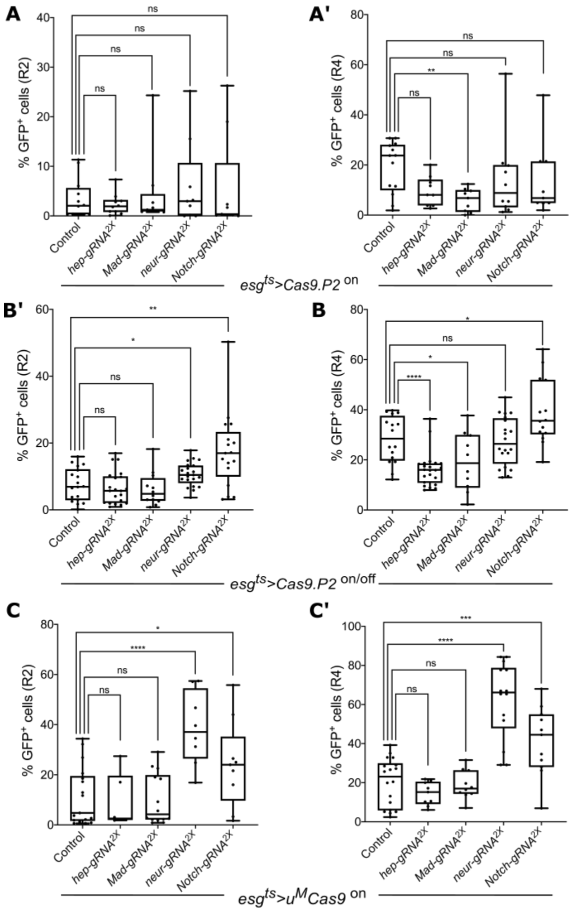

Figure S1. Boxplot representation of GFP⁺ cells after perturbation of the BMP, Notch and JNK pathways. (A-C) Quantification of GFP⁺ cells with either *Cas9.P2*^{on} (A, A’), *Cas9.P2*$^{on/off}$ (B, B’) and *u*^{M}*Cas9*^{on} (C, C’) with different *gRNAs* in both the R2 and R4 regions of the intestine.

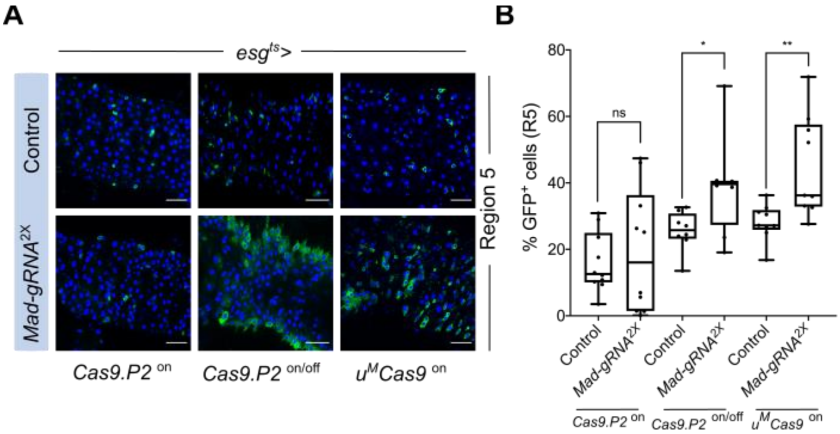

Figure S2. Quantification of GFP+ cells in the R5 region when Mad is perturbed with different Cas9 enzymes. Scale Bar: 30 µm.

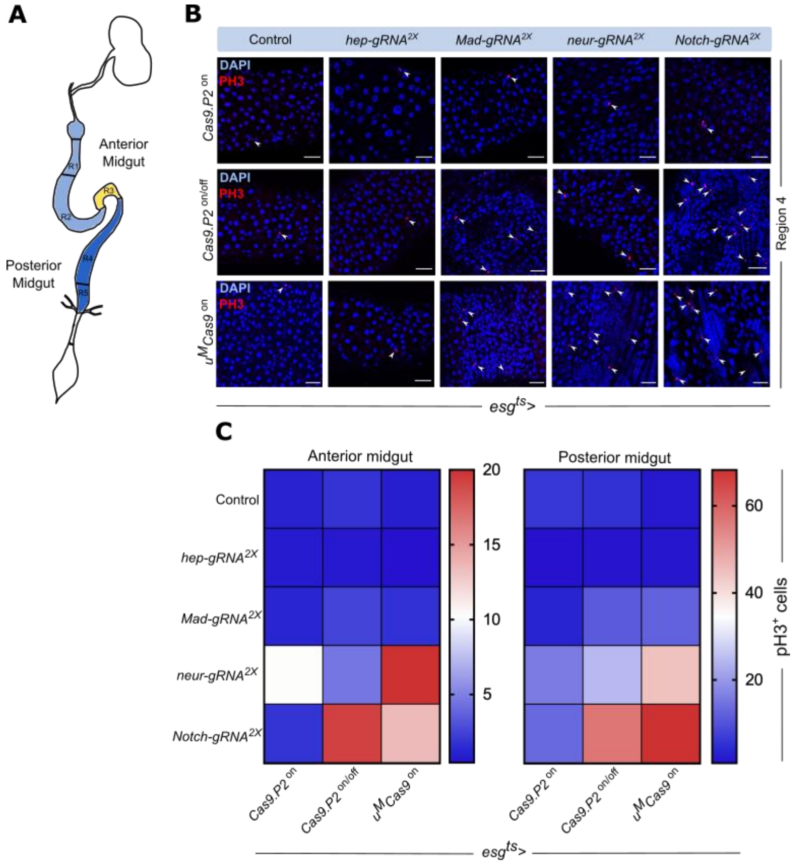

Figure S3. Cas9.P2$^{on/off}$ and u^{M}Cas9^{on} mediated perturbations of tumor suppressors increase the number of mitotic cells in the intestine. (C) Mitotic cells are marked with pH3 (red) and increase after *Cas9.P2$^{on/off}$* and *u^{M}Cas9^{on}* mediated perturbation of *Mad, neur* and *Notch*. (D) Heatmap showing the number of pH3+ cells in both the anterior and posterior regions of the intestine. Scale bar: 30 µm.

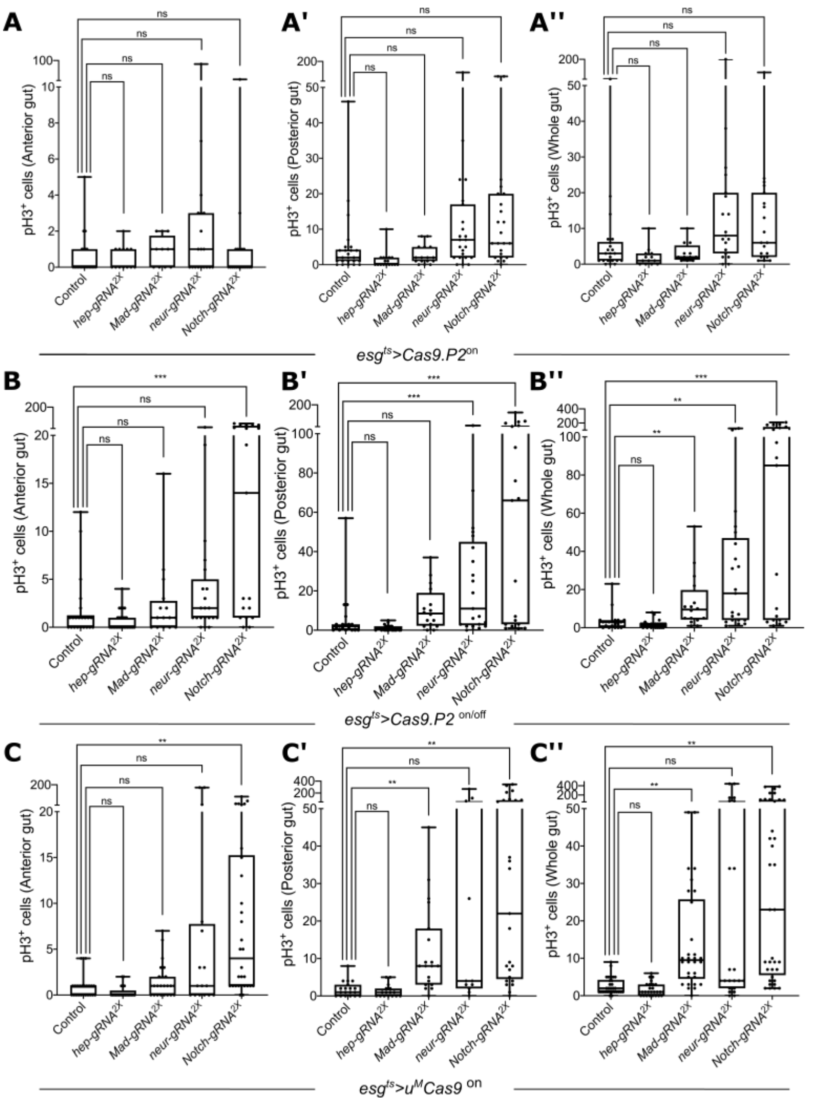

Figure S4. Boxplot representation of pH3+ cells after perturbation of the BMP, Notch and JNK pathways. (A-C) Quantification of pH3+ cells with either *Cas9.P2on* (A, A′, A′′), *Cas9.P2on/off* (B, B′, B′′) and *uMCas9on* (C, C′, C′′) with different *gRNAs* in the R2/R4 regions and the whole intestine.
